# Genetic diversity and connectivity of *Flaccisagitta enflata* (Chaetognatha: Sagittidae) in the tropical Atlantic ocean (northeastern Brazil)

**DOI:** 10.1371/journal.pone.0231574

**Published:** 2020-05-06

**Authors:** Danielle C. M. Melo, Simone M. A. Lira, Ana Paula B. Moreira, Lucas Freitas, Camilla A. D. Lima, Fabiano Thompson, Arnaud Bertrand, Alex C. Silva, Sigrid Neumann-Leitão

**Affiliations:** 1 Departamento de Oceanografia, Universidade Federal de Pernambuco, Recife, Brazil; 2 Programa de pós-graduação em Ecologia, Universidade Federal Rural de Pernambuco, Recife, Brazil; 3 Instituto de Biologia, Universidade Federal do Rio de Janeiro, Rio de Janeiro, Brazil; 4 Departamento de Genética, Evolução, Microbiologia e Imunologia, Universidade Estadual de Campinas, São Paulo, Brazil; 5 SAGE—COPPE, Centro de Gestão Tecnológica—CT2, Rio de Janeiro, Brazil; 6 Departamento de Pesca e Aquicultura, Universidade Federal Rural de Pernambuco, Recife, Brazil; 7 MARBEC, CNRS, Ifremer, IRD, Institut de Recherche pour le Développement (IRD), Université Montpellier, Sète, France; National Cheng Kung University, TAIWAN

## Abstract

The phylogeography of the holoplanktonic chaetognath *Flaccisagitta enflata* was investigated in the Tropical Western Atlantic (TWA). Considering the cosmopolitan range of this species and the fact that its entire life cycle is planktonic, the central hypothesis of this study is that *F*. *enflata* exhibits connectivity due to its high dispersal capacity, forming a panmictic population among the study sites. The evaluated areas included neritic (Port of Recife–PR, and Tamandaré - TA) and oceanic (Fernando de Noronha Archipelago—FN, Rocas Atoll—RA, Guará seamount—GS and Saint Peter and Saint Paul’s Archipelago—SPSPA) locations of the Brazilian Blue Amazon. We used COI gene sequences as molecular marker. Partial sequences (425 bp) were obtained for 116 specimens and employed to reconstruct the phylogeny, build an haplotype network, evaluate gene flow through a migration model, and estimate diversity indices, population structuring and demographic history. High levels of haplotype diversity (mean: 0.98) and moderate to high levels of nucleotide diversity (mean: 0.023) were observed. The phylogeny and the haplotype network topologies showed some geographic clustering, indicating local structuring in GS and PR. This finding was supported by the AMOVA high global Φst (0.033, significant) and some pairwise Φst comparisons (7 out of 15 were significantly >0). Significant differences suggested lower levels of connectivity when GS population was compared to those of FN and SPSPA; as well as when TA was compared to FN. These results might be related to particularities of the oceanic dynamics which rules the TWA, sustaining such dissimilarities. Structuring was also observed between PR and all oceanic locations. We hypothesize that the topography of the port inlet, enclosured by a reef barrier, may constrain the water turnover ratio and thus migration rates of *F*. *enflata* in the TWA. Accordingly, Migrate-N yielded a four metapopulations model (PR ⇌ TA ⇌ SPSPA+FN ⇌ GS+RA) as the best (highest probability; ~0.90) to represent the structuring of *F*. *enflata* in the TWA. Therefore, the null hypothesis of one randomly mating population cannot be accepted. The demographic evaluation demonstrated that the neutral hypothesis of stable populations may not be rejected for most of the locations. This work is the start point to broaden the knowledge on the phylogeography and population genetic structure of a numerically dominant species in the Western Atlantic, with key role in the marine trophic web.

## Introduction

The phylum Chaetognatha constitutes a group of small marine carnivores (2 to 120 mm) with broad distribution in coastal and oceanic regions of the world [1], occurring from the surface of the water column to depths into the abyssopelagic zone [2]. Chaetognaths are assiduous zooplankton predators, food items of a wide variety of taxa and producers of particulate organic matter [3, 4]. All species are hermaphrodites [5] and the life cycle of a large part of the group is holoplanktonic with direct development [1, 5], where from the hatching of the eggs emerge individuals very similar to adults with regard to body organization. The lifetime is variable and known for a few members of the phylum, with a maximum estimate of approximately 15 months for a species investigated in shelf waters of the Atlantic Ocean [6].

Holoplanktonic species are generally characterized by large population sizes, high fecundity rates and broad dissemination by marine currents [7]. These aspects can also be applied to Chaetognatha [e.g. 6, 8, 9] making them an interesting model to understand dispersal processes and genetic connectivity of plankton among geographically distant environments. Recent research based on molecular genetics have demonstrated, for example, important connections between occurrence ranges of species and the gene flow in the pelagic zone [9–11], as well as the existence of cryptic complexes in different oceanic regions [9, 10, 12].

Among the cosmopolitan species of Chaetognatha, *Flaccisagitta enflata* (Grassi, 1881) was selected as model for this study due to its extensive horizontal distribution and high abundance in marine plankton. This species can reach lengths of up to 25 mm in the adult stage and presents a typical occurrence in epipelagic waters of the tropical and subtropical regions throughout the world [2, 13–15], including neritic and oceanic areas of northeastern Brazil, where it is usually the most abundant species of the phylum [16–18]. Its distribution based on conventional taxonomic identification is, therefore, well documented in the literature. However, specific data on the genetic diversity and connectivity of *F*. *enflata* remains scarce [19], since most molecular studies involving this species are directed at investigating the phylogenetic position or evolutionary history of Chaetognatha [eg. 20–22].

Previous studies with different molecular markers demonstrated that species with broad distribution and at least one planktonic larval stage are characterized by high levels of gene flow and form a single population between the Brazilian coast and oceanic islands of the Tropical Western Atlantic (TWA) (Echinodermata [23], Polychaeta [24] and reef fish [25]). Marine currents often favor this scenario, providing an effective transportation for planktonic organisms contributing to their long-range dispersal [26–28]. Moreover, biological factors such as species ecology and behavior, in association with their interactions with the oceanographic environment, are important determinants of connectivity [29] or genetic isolation among populations [eg. 30]. Although the gene flow is suggestively high among holoplankton even at oceanic scales [eg. 31–33], some studies have reported varied levels of genetic structure for widely distributed zooplanktonic species, investigated from macro to meso geographical scales [9, 29, 34–36].

Such issues are often clarified by the analysis of mitochondrial DNA (mtDNA) in population studies. MtDNA provides a series of advantages, such as a faster evolutionary rate (both in terms of mutation rate and rate of genetic drift) compared to the nuclear genome and the presence of genes considered highly informative for the diagnosis of eukaryotes [37, 38]. Its *cytochrome oxidase subunit I* (COI) gene, for example, has been proposed as the universal molecular system (barcode) for species identification [39], being already successful in clarifying the phylogeography of Chaetognatha [11, 12, 40].

Based on these considerations, we aimed at (i) determining the level of genetic diversity of *F*. *enflata;* (ii) describing the genetic connectivity pattern exhibited by this species between the neritic and oceanic regions of northeastern Brazil (TWA), and (iii) providing an overview of its demographic history; employing COI gene sequences analysis. Considering the cosmopolitan range of this species and that its entire life cycle is planktonic, the central hypothesis of this study is that *F*. *enflata* exhibits connectivity due to its high dispersal capacity, forming a panmictic population among the study sites; here comprised in a geographic small- (tens to hundreds of kilometers) to meso-scale (thousands of kilometers) approach. This hypothesis can also be sustained by virtue of low mutation rates observed for Chaetognatha along an extensive evolutionary history, probably originated at the Cambrian onset (~ 540–520 Myr ago) [41].

This work provides original data (including genetic) on a species that is both highly abundant and prevalent in the holoplankton of Brazilian waters thus playing a major ecological role.

## Materials and methods

### Study areas

**Fig 1:**
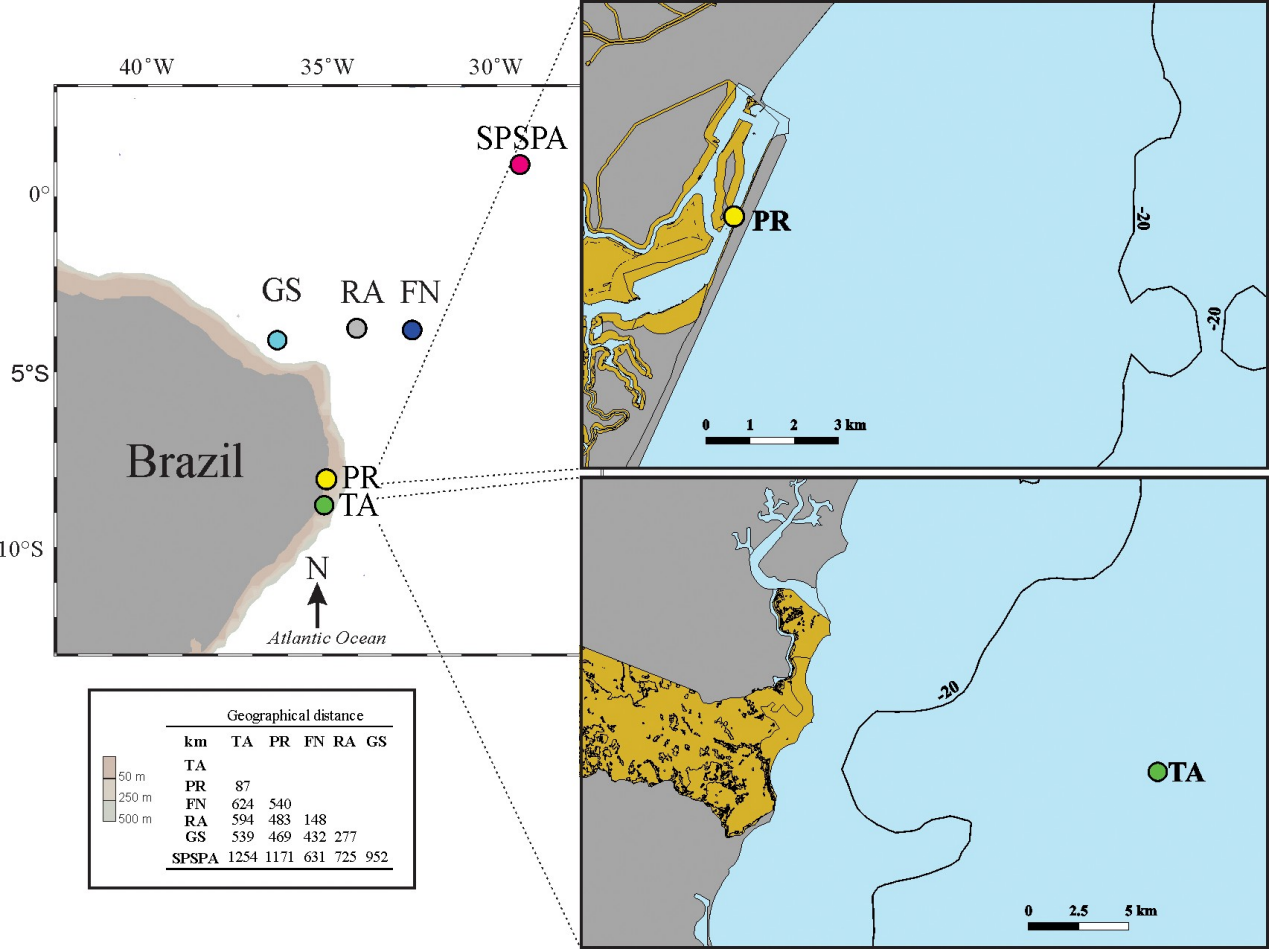
Sampling locations. The colored circles represent the areas included in the present study. Green: Tamandaré (TA); yellow: Port of Recife (PR); light blue: Guará seamount (GS); grey: Rocas Atoll (RA); dark blue: Fernando de Noronha Archipelago (FN); pink: Saint Peter and Saint Paul’s Archipelago (SPSPA). The sampling points in PR and TA were highlighted for better visualization. Below are the geographical distances between the locations evaluated. Map generated in Ocean data View ODV 5.1 (https://odv.awi.de/) and Quantum Gis v. 3.4 (www.qgis.org).

The study areas included neritic (Port of Recife—PR, and Tamandaré continental shelf—TA) and oceanic (Fernando de Noronha Archipelago—FN, Saint Peter and Saint Paul’s Archipelago—SPSPA, Rocas Atoll- RA and Guará seamount—GS) locations in northeastern Brazil (TWA) (Fig 1).

PR (08°03.4’S; 34°52.1’W) and TA (8°47’20”S; 35°06’45”W) are respectively part of the central and southern neritic zones of the state of Pernambuco. PR is highly impacted by human activities, such as the discharge of domestic and industrial waste [42]. TA integrates the Coral Coast Preservation Area, one of the largest marine conservation units of Brazil. Evidences of eutrophication have not been reported for this site [43].

The volcanic islands of FN (03°51’S; 32°25’W) and RA (03°50’S; 33°49’W) integrate the Fernando de Noronha Mountain Chain, which extends from the Brazilian continental shelf to the FN archipelago, and also includes several seamounts, as Guará (4°5’24.79”S; 36°18’03.42”W) [44]. FN is instituted as National Marine Park and State Environmental Protection Area, located 345 km off the coast of Brazil and 148 km apart from RA. This latter is located 260 km off the coast of Rio Grande do Norte state and recognized as the first biological reserve in Brazil [45]. North of the Equator (0°55’06”N; 29°20’48” W), the SPSPA comprises a group of rocky islands on top of Mid Atlantic Ridge tectonic fault, where the depth range is 4–5000 meters [46]. This archipelago is located 1010 km off the coast of Brazil and 610 km distant from FN [47], comprising the FN—RA—SPSPA Environmental Protection Area [45].

### Sampling

Plankton samples from six sites in the TWA (Fig 1, S1 Table) were collected using standard plankton nets with 300 and a 500 μm mesh sizes. In the neritic area, horizontal hauls were used to sample the surface layer. Oceanic sites were sampled during two expeditions, through of research vessels Transmar I (FN and ASPSP), in the scope of the project “Plankton community in the Saint Peter and Saint Paul’s Archipelago and its association with physical mechanisms: vertical distribution of diversity and productivity”; and Antea (Rocas Atoll and Guará seamount), in the scope of the project “Acoustics along the Brazilian Coast 2”. In these locations, surface horizontal hauls were performed, as well as oblique hauls out in the depth-layer of 0–200 meters, whenever possible.

In the field, samples were washed in sterile 3% saline solution and subsequently fixed in 96% ethanol. In the laboratory, the chaetognaths were quickly separated from the material obtained and the species *F*. *enflata* was identified based on specialized literature [1]. Tissue samples (up to 25 mm^3^) were then removed from the best-preserved individuals and with no apparent stomach contents, through dissection with previously sterilized disposable blades. In total, tissue samples from 36 individuals from the PR, 16 from TA, 19 from FN, 12 from the GS, seven from RA and 26 from SPSPA were conserved in 100% ethanol and at a temperature of 4°C until the DNA extraction step.

### Sampling permits

Ministério do Meio Ambiente (MMA): Instituto Chico Mendes de Conservação da Biodiversidade (ICMBio—Number 17689) and Sistema de Autorização e Informação de Biodiversidade (SISBio—Number 47270–5).

### DNA barcoding and phylogeny

Total DNA was obtained using the *Blood and Tissue* extraction kit from Qiagen, following the manufacturer’s protocol. The universal primers for COI amplification: LCO1490 (5’- GGT CAA CAA ATC ATA AAG ATA TTG G—3’) and HCO2198 (5’- TAA ACT TCA GGG TGA CCA AAA AAT CA—3’) were used [48]. PCRs were performed in 20 μl, consisting of 10 μl of Master Mix Go Taq G2 C (Promega), 5 pmol of each primer, and 20 to 50 ng of extracted DNA. The reaction protocol involved an initial denaturation step at 95°C for 1 minute; followed by 35 cycles of denaturation at 94°C for 30 seconds, annealing at 52°C for 40 seconds and extension at 72°C for 1 minute; with a final extension step at 72°C for 5 minutes performed at the end of the last cycle (modified from [48]). Products were purified, sequenced and edited to generate consensus sequences, which were compared to the Genbank database to retrieve the most similar sequences, as described in [49].

Consensus sequences were exported to AliView v. 1.18.1 [50] and translated to inferred amino acids to verify that they translated correctly. The set of amino acid sequences were then aligned using the Multiple Sequence Comparison by Log-Expectation (MUSCLE) tool [51] in AliView and returned to DNA format. The alignment was manually edited and primer sequences were removed. Sequences generated in this work were deposited under the BARCODE section of GenBank along with metadata (Accession Numbers MH244934- MH245049) (S1 Table). For reference, 15 COI sequences from GenBank were added to the phylogenetic analysis (S2 Table). To investigate the evolutionary history, a model of DNA sequence evolution was selected using the Smart Model Selection (SMS) [52] under Akaike’s Information Criterion (AIC). The General Time Reversible (GTR) model was selected, with an estimated proportion of DNA sites invariant (I; 0.465 sites), and mutation rates among sites following a gamma distribution (G) (GRT +I +G) [53]. A Maximum Likelihood (ML) tree was generated with PhyML 3.1 [54] starting from a neighbor-joining tree. The support for the nodes was assessed using the approximate likelihood ratio test for branches (aLRT) [55] and the bootstrap test (1000 repetitions). The tree was visualized using iTOL v.3 [56] and included 131 sequences with 425 nucleotide positions.

### Population genetics

The following genetic diversity indices: number of haplotypes (H), haplotype diversity (Hd), number of polymorphic sites (Nps), mean number of nucleotide differences (MnNd) and nucleotide diversity (π) were obtained from the Arlequin v.3.5 [57] and DnaSP v. 5.0 [58] programs.

An haplotype network was constructed to depict the general genealogy patterns at the intraspecific level. For such, the PopArt v.1.7 program (www.popart.otago.ac.nz/index.shtml) was used, employing the median-joining criterion.

Population genetic structuring was investigated with the Bayesian Analysis of Population Structure (BAPS) 5.0 program [59], which identifies and groups genetically similar individuals in panmictic groups, henceforth denominated haplogroups. The parameters considered were “analysis of genetic mixture with linked loci or sequences” and “population mixture” estimated at each 10,000 generations per individual.

Within-population and between-population structure was examined through an analysis of molecular variance (AMOVA) [60] and genetic pairwise differentiation from the fixation index (ΦST) [61], both based on the F statistic and using 10,000 permutations in Arlequin v.3.5 [57]. Due to the significant structuring values demonstrated by general AMOVA and some pairwise comparisons, new AMOVA tests were performed considering different groupings scenarios.

In order to find the most appropriate migration model (MM) to characterize the population structure in the region, different tests were performed in the Migrate-N v.4.4 software [62, 63]. For each model we performed three independent Markov Chain Monte Carlo (MCMC) runs. Each MCMC run consisted in the analysis of four distinct chains with different temperatures (1,000,000; 3; 1,5 and 1) to allow a better exploration of the parametric space. MCMC runs were sampled each 250^th^ generation to a total of 20,000,000 generations. The parameters were estimated after a burn-in of 8,000,000 generations in each run. The probability of each model was calculated following Migrate-N tutorial using the average log marginal likelihood of three independent runs, as shown in Table 4. These log marginal likelihoods were estimated using the Bezier-Corrected model because it outperformed other models in simulations [63, 64]. First, all MM log marginal likelihoods were subtracted from MM log marginal likelihood average values, generating a list of values. Each element of this list was then exponentiated and their individual results were summed to obtain the equivalent denominator. Finally, the probability of each MM was obtained dividing each element from the exponentiated list by the denominator. We created 11 metapopulation models (migration models, MMs, Table 4) to compare based in previous studies of connectivity in the TWA and of Chaetognatha in the North Atlantic; in the distances between sites and regional structures (Fig 1); in the results of the AMOVA tests, and also in the currents, according to the circulation model (Fig 4) built as the description hereafter. MM1 represented panmixia (connectivity in the area was described in [23, 24, 25]). We modeled 2 populations based in the AMOVA results and presuming a barrier to gene flow being (a) the reef line parallel to PR (MM2.1 and MM2.3); or (b) the local circulation particularities of the Brazil Current system (unidirectional northwestwards); and of the South Equatorial Current system with a superficial partial retroflection in FN area, and with the undercurrent drifting away from GS (MM2.2). Three populations were modeled (a) grouping sites by distance (MM3.1); (b) based in the general AMOVA results (MM3.2), and (c) a variation of MM3.1 and 2 (MM3.3). Two 4 populations’ models (a) detached GS from its group in MM3.1, based in the AMOVA results and local currents (explained for MM2.2), and (b) detached TA from its group in MM3.3 based in the AMOVA results and the regional structure (explained for MM2.1 and 3). The duo TA+PR of MM4.1 was split to create a 5 populations model (MM5) based in the AMOVA results and the regional structure. MM6 assumes gene flow occurs between sites that neighbor each other in the specification (Table 4). The significance of the best model (highest probability) was tested by comparing the mean marginal likelihood with the second best model using a *t* test. A *p*-value > 0.05 indicated ambiguity.

The demographic history of *F*. *enflata* in each locality was inferred using (i) Tajima’s *D*, a statistical method that tests the neutral mutation hypothesis by DNA polymorphism [65]; and, (ii) Fu’s *Fs*, a method that tests the neutrality of mutations against population growth, hitchhiking and background selection [66]. Typically, assuming effective neutrality (null hypothesis) allows for demographic inferences. For both indices, significant positive/negative values are representative of population reduction/expansion. Indices were calculated through Arlequin v.3.5 [57] applying 10,000 permutations to determine the statistical significance.

### Circulation model

To complement the results and provide a better basis for discussing connectivity aspects, the outputs of an oceanic dynamic model approach was implemented. The model was provided by Mercator Ocean NEMO configuration with a 1/12° high resolution centered over the Atlantic (https://www.mercator-ocean.fr/), which uses a 0.25° ORCA grid [67]. The water column was discretized into 50 vertical levels, including 22 levels within the upper 100 m, with 1 m resolution at the surface to 450 m resolution at the bottom. The ocean model is forced by atmospheric fields from the European Centre for Medium-Range Weather Forecasts Integrated Forecast System (ECMWF-IFS) at 3 h resolution to reproduce the diurnal cycle. The system was initialized in fall 2006, using temperature and salinity profiles from the EN4 climatology [68]. Observing System Experiments (OSEs) were conducted with the Mercator Ocean global ocean forecast systems. Similar Observing approaches are generally used to evaluate observation networks in the ocean data assimilation community of GODAE Ocean View [69].

The numerical velocity fields data corresponded to the same periods of the acoustics along the Brazilian Coast survey, and were used to associate the large-scale circulation with the biological observations that span a relatively limited area.

## Results

After curation and alignment, 425 bp fragments of the COI mitochondrial gene from 116 specimens of *F*. *enflata* were selected for analysis. The genetic indices demonstrated high and uniform haplotype diversity values; whereas more heterogeneous values, and moderate to high, were found for nucleotide diversity (Table 1). Mean haplotype diversity was 0.97, encompassing a minimum of 0.90 for PR to a maximum of 0.99 for TA and GS. Nucleotide diversity ranged from 0.018 (at three of the six locations: PR, FN and GS) to 0.035 (RA), with an overall mean of 0.023 including all the six areas. In total, 61 COI haplotypes were detected (Table 1). Amongst the 425 sites, 87 were polymorphic (20.47%), with the range of polymorphic sites varying from 32 (FN) to 43 (TA). The mean number of nucleotide differences comprised a minimum of 7.39 (PR) to 14.67 (RA) (Table 1).

**Table 1:** Molecular diversity indices for the COI region of mtDNA of *Flaccisagitta enflata*, from neritic and oceanic locations in Tropical Western Atlantic.

GENETIC INDICES	All samples	SAMPLING LOCATIONS					
		TA	PR	SPSPA	FN	GS	RA
N	116	16	36	26	19	12	7
H	61	15	20	18	12	11	6
Hd	0.97	0.99	0.90	0.95	0.94	0.99	0.95
Nps	87	43	41	41	32	34	37
MnNd	9.57	8.21	7.39	11.85	7.60	7.55	14.67
π	0.023	0.019	0.018	0.028	0.018	0.018	0.035

Locations abbreviations: TA—Tamandaré; PR—Porto of Recife; SPSPA—Saint Peter and Saint Paul’s Archipelago; FN—Fernando de Noronha Archipelago; GS—Guará Seamount; RA—Rocas Atoll. Genetic Indices abbreviations: N—sample size; H—number of haplotypes; Hd—haplotype diversity; Nps—number of polymorphic sites; MnNd—mean number of nucleotide differences; and π - nucleotide diversity.

**Fig 2:**
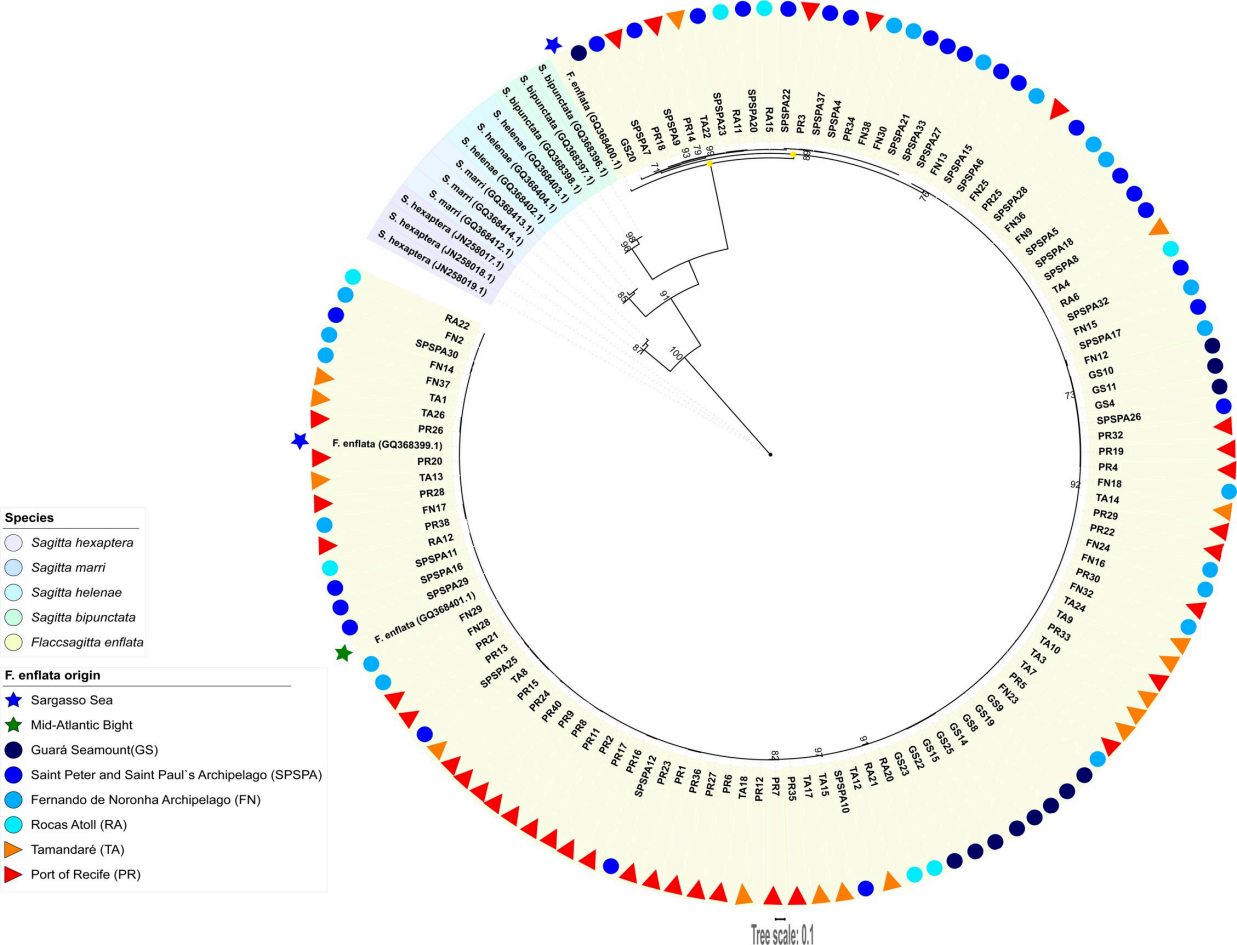
Gene tree for COI showing topology based on Maximum-Likelihood (ML) criterion. Node support values are indicated by whole numbers > 70 and represent percentages. Scale bar denotes distance along branches. Sequences retrieved from GenBank are identified by the accession numbers. Sequences from this study are identified by location abbreviations and sample number (S1 Table). The main nodes of the *Flaccisagitta enflata* branch are depicted with circles: *F*. *enflata* species branch, empty; single-haplotype branch, red filled; 19-haplotypes branch, yellow filled; others, black filled. Branches of the 19-haplotypes cluster are colored yellow.

The optimal phylogenetic tree presented long branches separating the species whereas short branches clustered each species (Fig 2). The monophyletic *F*. *enflata* group bifurcated into one branch comprising a single sequence from the Sargasso Sea (SS; GQ368400.1), and another with all the remainders. The latter multiple branch further bifurcated clustering 19 sequences (smaller branch) from the present study apart from all the others (larger branch), including one from the SS and another from the Mid-Atlantic Bight (MAB; North Atlantic). The six locations sampled in this study were represented in both branches stemming from this bifurcation. The 19-sequences smaller branch harbored only one haplotype from GS. Additionally, PR haplotypes appeared somewhat clustered (Fig 2).

**Fig 3:**
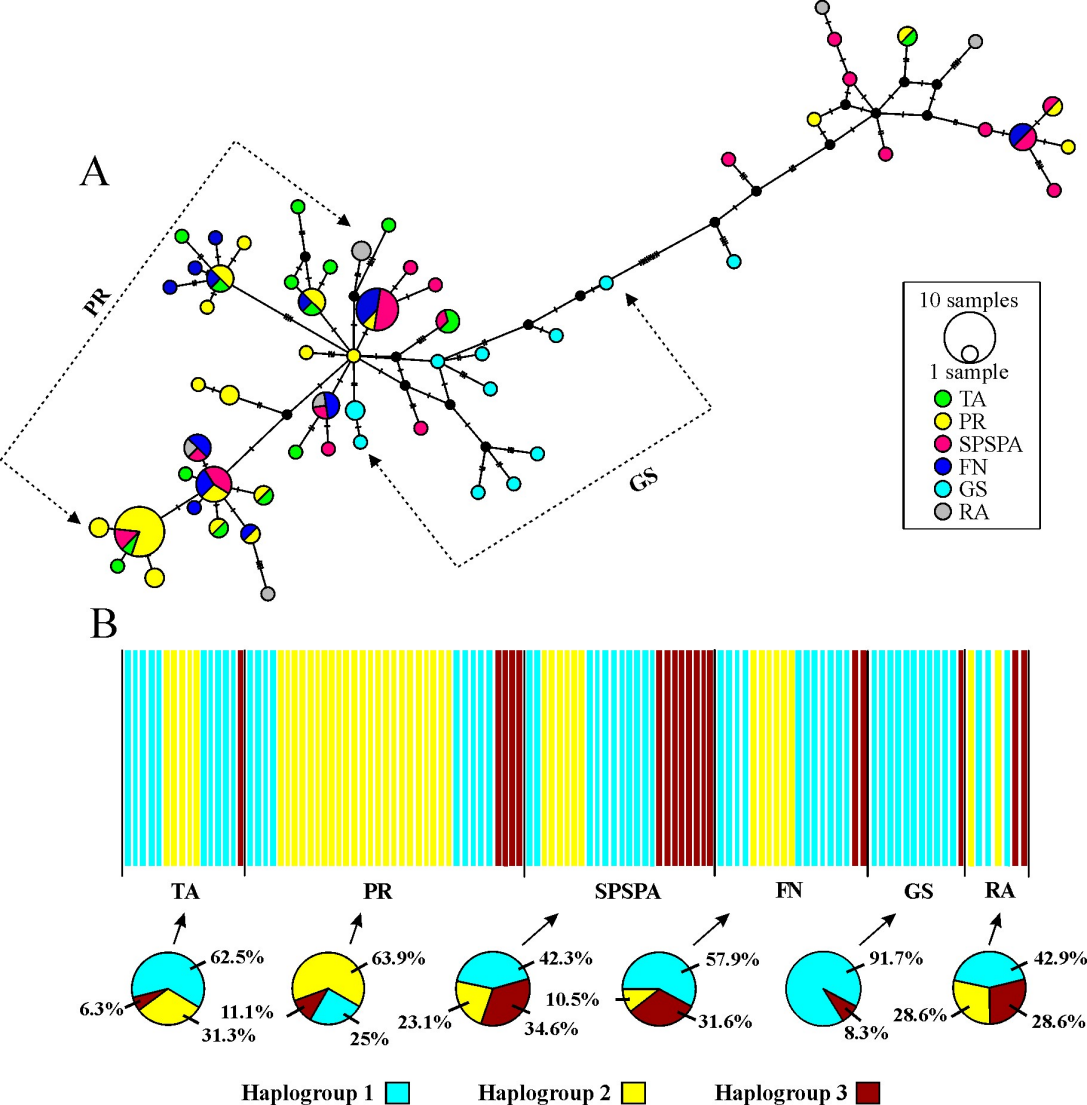
Haplotype network and Bayesian Analysis of Population Structure (BAPS) based on the COI region of mtDNA from *Flaccisagitta enflata* from neritic and oceanic locations in Tropical Western Atlantic. A. Haplotype network generated for 61 haplotypes from 116 sequences of the species. The circles’ area is proportional to the haplotype’s frequency (1 and 10 samples). The colors indicate location. Dotted arrows represent the haplotype groups of PR and GS B. BAPS. The colors represent different haplogroups and the circular pizza graphs represent the distribution of the haplogroups per location. Abbreviations: TA—Tamandaré; PR—Port of Recife; SPSPA—Saint Peter and Saint Paul’s Archipelago; FN—Fernando de Noronha Archipelago; GS—Guará seamount; and RA—Rocas Atoll.

Overall, the patterns and the mild clustering trends were replicated in the haplotype network (Figs 2 and 3A). The network topology exhibited two subgroups separated by ten mutational steps (Fig 3A). Among the 61 haplotypes recorded, 14 were shared among the different study areas, the most frequent of which was H11 (12.07% - 1 individual from TA, 11 from PR and 2 from SPSPA); followed by H25 (8.62% - 1 individual from PR, 5 from SPSPA and 4 from FN) and H23 (7.76% - 2 individuals from PR, 03 from SPSPA and 2 from FN). Subgroup 1 (smaller) was formed by 3 shared haplotypes (H13, H27 and H25) and 11 single-frequency haplotypes, representatives of the 19 sequences clustered in the *F*. *enflata* smaller branch of the tree. Subgroup 2 joined the 47 remaining haplotypes, including the 11 shared and 36 single-frequency haplotypes from the neritic and oceanic locations (Fig 3A).

The BAPS analysis detected three haplogroups (k = 3), which were present in five out of the six locations. GS was the only area to exhibit only two haplogroups, with > 90% of the individuals affiliated to haplogroup 1 (Fig 3B). Haplogroup 1 was dominant in all locations, with the exception of PR, where the haplogroup 2 prevailed (63.89%). These two haplogroups (1 and 2) were mainly represented within subgroup 2 in the haplotype network (Fig 3A and 3B). All individuals were correctly assigned to their respective haplogroups (p > 0.05).

### Population structure

The AMOVA general test (Table 2) showed a significant high global level of genetic structuring when testing all locations (Φst = 0.033; p < 0.001), with a much higher percentage of molecular variation within populations (96.7%) than among them (3.3%). When testing for specific differences, significant structuring (in both Φst and Φsc) was observed between neritic (PR, TA) and insular locations (all the others); as well as between PR vs. all other locations, and TA vs. all other locations (Table 2)

**Table 2:** Analyses of molecular variance (AMOVA) based on the COI region of mtDNA *Flaccisagitta enflata*, from neritic and oceanic locations in Tropical Western Atlantic.

COMPARISONS/SOURCE OF VARIATION	d.f.	Percentage of Variation	Φ Statistic
**All Locations**			
Among populations	5	3.34	Φst = 0.033*
Within populations	110	96.66	
**Neritic (TA, PR) vs. Insular (FN, RA, GS, SPSPA) Locations**			
Among groups	1	2.36	Φct = 0.023
Among populations within groups	4	1.86	Φsc = 0.020*1
Within populations	110	95.78	Φst = 0.042*
**PR vs. All Other Locations (TA, FN, RA, GS, SPSPA)**			
Among groups	1	2.62	Φct = 0.026
Among populations within groups	4	1.91	Φsc = 0.020*
Within populations	110	95.47	Φst = 0.045*
**TA vs. All Other Locations (PR, FN, RA, GS, SPSPA)**			
Among groups	1	-1.56	Φct = -0.015
Among populations within groups	4	3.87	Φsc = 0.038*
Within populations	110	97.69	Φst = 0.023*
**GS vs. All Other Locations (TA, PR, FN, RA, SPSPA)**			
Among groups	1	0.88	Φct = 0.033
Among populations within groups	4	3.13	Φsc = 0.001
Within populations	110	95.98	Φst = 0.034
**GS vs. Other Oceanic Locations (FN, RA, SPSPA)**			
Among groups	1	3.30	Φct = 0.033
Among populations within groups	2	0.14	Φsc = 0.001
Within populations	60	96.56	Φst = 0.034
**GS vs. FN, SPSPA**			
Among groups	1	5.17	Φct = 0.052
Among populations within groups	1	-1.71	Φsc = -0.018
Within populations	54	96.54	Φst = 0.035

Abbreviations: d.f—degrees of freedom; TA—Tamandaré; PR—Port of Recife; SPSPA—Saint Peter and Saint Paul’s Archipelago; FN—Fernando de Noronha Archipelago; GS—Guará seamount; and RA—Rocas Atoll.

* *p* < 0.001

Pairwise Φst comparisons (Table 3) detected statistically significant genetic differences between PR and all oceanic locations (SPSPA, FN, GS and RA). In addition, significant differences were observed between GS compared to SPSPA and FN; as well as between TA and FN. In this second round, 7 out of the 15 comparisons were significant.

**Table 3:** Pairwise fixation indices (Φst) based on the COI region of mtDNA between individuals of *Flaccisagitta enflata*, from neritic and oceanic locations in Tropical Western Atlantic.

LOCATION	TA	PR	SPSPA	FN	GS	RA
**TA**	**-**					
**PR**	0.024	-				
**SPSPA**	0.018	0.038*	-			
**FN**	0.027*	0.061*	-0.018	-		
**GS**	0.012	0.060*	0.032*	0.038*	-	
**RA**	0.026	0.076*	0.036	0.024	0.030	-

Abbreviations: TA—Tamandaré; PR—Port of Recife; SPSPA—Saint Peter and Saint Paul’s Archipelago; FN—Fernando de Noronha Archipelago; GS—Guará seamount; and RA—Rocas Atoll.

*p < 0.05

Migrate-N inferred the highest probability of ~0.90 to the 4 populations scenario MM4.2 (PR ⇌ TA ⇌ SPSPA+FN ⇌ GS+RA). This model defined 2 neritic groups (PR, TA) and 2 oceanic groups (SPSPA, FN and GS, RA). The model with the second highest probability of ~0.05 was that of 2 populations MM2.3 (PR+TA ⇌ SPSPA+FN+RA+GS) (Table 4). This model grouped neritic (PR, TA) and insular locations (SPSP, FN, RA, GS); groups also distinguished by general AMOVA (Table 2). The *t* test *p*-value was > 0.05 when these models (MM4.2 and MM2.3 populations) were compared suggesting some level of ambiguity.

**Table 4:** Log marginal likelihood for distinct runs and migration models (MMs) with the respective average and relative probability based on the COI region of the mtDNA of *Flaccisagitta enflata*. MMs with > 0 probability in bold.

MODEL	METAPOPULATIONS	LOG MARGINAL LIKELIHOOD			MODEL PROBABILITY (%)	
		Run1	Run2	Run3	Mean	
MM1	TA+PR+SPSPA+FN+RA+GS	-2120.2	-2116.59	-2118.89	-2118.56	6.59 x 10^−22^
MM2.1	TA ⇌ PR+SPSPA+FN+RA+GS	-2091.97	-2090.2	-2096.17	-2092.78	1.03 x 10^−10^
MM2.2	GS ⇌ TA+PR+SPSPA+FN+RA	-2066.94	-2077.19	-2076.14	-2073.42	0.026
**MM2.3**	**PR+TA ⇌ SPSPA+FN+RA+GS**	**-2077.72**	**-2068.08**	**-2058.59**	**-2068.13**	**5.25**
MM3.1	TA+PR ⇌ SPSPA ⇌ FN+GS+RA	-2060.98	-2079.98	-2089.93	-2076.96	7.65 x 10^−04^
MM3.2	PR ⇌ TA+FN+SPSPA+RA ⇌ GS	-2081.64	-2078.34	-2080.44	-2080.14	3.19 x 10^−05^
MM3.3	PR ⇌ TA+FN+SPSPA ⇌ GS+RA	-2083.34	-2073.24	-2092.7	-2083.09	1.66 x 10^−06^
MM4.1	TA+PR ⇌ SPSPA ⇌ FN+RA ⇌ GS	-2092.05	-2078.05	-2074.28	-2081.46	8.53 x 10^−06^
**MM4.2**	**PR ⇌ TA ⇌ SPSPA+FN ⇌ GS+RA**	**-2071.03**	**-2052.34**	**-2072.5**	**-2065.29**	**89.84**
MM5	TA ⇌ PR ⇌ SPSPA ⇌ FN+RA ⇌ GS	-2082.91	-2069.77	-2070.04	-2074.24	0.012
**MM6**	**TA ⇌ PR ⇌ SPSPA ⇌ FN ⇌ RA ⇌ GS**	**-2077.89**	**-2062.72**	**-2064,02**	**-2068.21**	**4.86**

### Demographic history

Tajima’s *D* and Fu’s *Fs* estimates demonstrated negative values in most cases, however statistical significance was only detected for Fu’s value of TA (*p* < 0.02) (Table 5). Exceptions were found for SPSPA and RA, which presented, non-significant positive values for Tajima’s index (*D* = 0.39, *p* > 0.05) and Fu’s index (*Fs* = 0.81; *p* > 0.02), respectively (Table 5).

**Table 5:** Demographic indices based on the COI region of the mtDNA of *Flaccisagitta enflata*, from neritic and oceanic locations in Tropical Western Atlantic.

DEMOGRAPHIC INDICES	TA	PR	SPSPA	FN	GS	RA	Mean
Tajima index (D)	-1.54	-0.91	0.39	-0.68	-1.49	-0.16	-0.73
*p* of D	0.05	0.18	0,71	0.26	0.06	0.45	0.28
Fu index (Fs)	-6.89	-4.37	-2,53	-1.33	-3.82	0.81	-3.02
*p* of Fs	0.003*	0.07	0,16	0.03	0.03	0.57	0.18

Abbreviations: TA—Tamandaré; PR—Port of Recife; SPSPA—Saint Peter and Saint Paul’s Archipelago; FN—Fernando de Noronha Archipelago; GS—Guará seamount; and RA—Rocas Atoll.

*p < 0.02

### Circulation model

**Fig 4:**
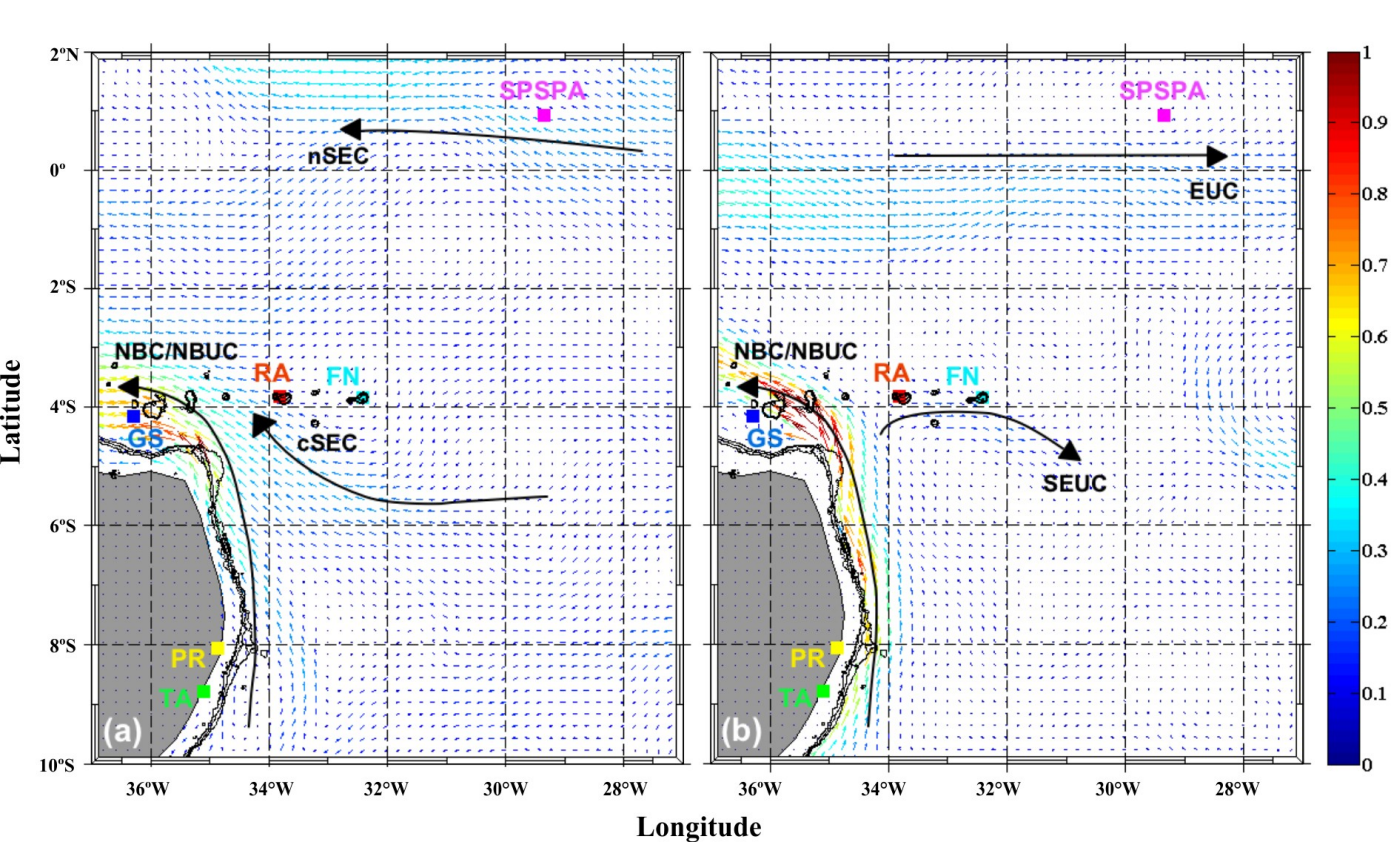
Horizontal distribution of current velocity from Mercator model results along 0–50 m (A) and 150 m (B). Chaetognaths sampling locations are indicated by colored squares—green: Tamandaré (TA); yellow: Port of Recife (PR); light blue: Guará seamount (GS); gray: Rocas Atoll (RA); dark blue: Fernando de Noronha Archipelago (FN); pink: Saint Peter and Saint Paul’s Archipelago (SPSPA). Main currents that surround in the areas: cSEC = central South Equatorial Current; nSEC north South Equatorial Current; NBC/NBUC = North Brazil Current/North Brazil Undercurrent system; EUC = Equatorial Undercurrent; SEUC = South Equatorial Undercurrent. The black lines represent the isobaths 50 m, 500 m and 1000 m.

Numerical model outputs for the first 50 m of the water column and 150 m depth were used to analyze the surface circulation along the coast and oceanic waters of northeastern Brazil. Currents dynamic of the TWA is dominated by the North Brazil Current/North Brazil Undercurrent (NBC/NBUC) system, which is mainly controlled by the different branches of the South Equatorial Current (SEC) (Fig 4). Characteristics of the regional Currents can be obtained from a realistic numerical model.

In the top 50 meters (Fig 4A), modeled results indicated the presence of a continuous northwestward flow near and along the shelf break corresponding to the NBC/NBUC system. Between 5–6° S this flow intensifies, with mean velocity reaching a maximum of 1 m.s^-1^ around the GS. At the same depth, the influence of the central branch of the SEC (cSEC) was registered, with an average velocity of 0.3 m.s^-1^, transporting waters from eastern tropical Atlantic to the oceanic region around RA and FN. The model also indicates the presence of westward flows associated with the north branch of the SEC (nSEC), which are located in the same region of the SPSPA. Likewise, the model results for 150 m depth (Fig 4B) depict the northwestward transport provided by the NBC/NBUC along the shelf break (parallel to the Northeastern Brazilian shelf), associated with relatively high velocity (0.6–1.0 m.s^-1^). The model also recorded the presence of the South Equatorial Undercurrent (SEUC), transporting waters eastward from the near shelf break, reaching the regions around RA and FN. These results suggested the presence of the Equatorial Undercurrent (EUC) around SPSPA, which is in good agreement with an eastward deviation of the waters coming from the western tropical Atlantic, off the coast of Brazil.

## Discussion

### Genetic diversity and connectivity

In the present investigation, the COI molecular marker satisfactorily confirmed the identity and evaluated the diversity and phylogeographic aspects of the species *F*. *enflata*. This is the first study of its kind for the TWA, where nothing was known regarding the molecular genetics and phylogeography of this predominant zooplanktonic species. Its high frequency in both space and time [eg. 18] makes this chaetognath a key species in the marine trophic chain, exerting a relevant influence on the zoo- and ichthyoplanktonic communities. Our results suggest that even an holoplanktonic and cosmopolitan species may deviate from the received wisdom of genetic homogeneity in a regional approach.

In general *F*. *enflata* displayed high levels of genetic diversity, which is frequently associated with large population sizes of zooplankton [31] and stable environmental conditions for a long evolutionary period [70]. Similar nucleotide diversity data using the COI gene was described for populations of the harpacticoid copepod *Macrosetella gracilis* in the Pacific and Atlantic oceans (π = 0.010 and 0.044, respectively) [71]. However, haplotype diversity for *M*. *gracilis* was lower compared with that found in the present study (Hd = 0.69 [Atlantic] to 0.96 [Pacific]). Other chaetognaths also presented lower diversity based on COI, such as *Eukrohnia fowleri* in the Atlantic (π = 0.00 and Hd = 0.12) [72] and *E*. *hamata* in three oceans (Atlantic: π = 0.005 and Hd = 0.833; Arctic: π = 0.010 and Hd = 0.844; Antarctic: π = 0.004 and Hd = 0.542) [11].

In the global ocean, it is common that widely distributed planktonic species exhibit a high dispersion capacity [31, 73]. This aspect was here confirmed through the phylogeny and haplotype network topologies, which identified a weak geographic clustering. The separation between the haplotypes defined by a few mutational steps was also indicative that the dispersion occurred over long distances, which may explain the presence of shared haplotypes among very distant locations (up to 1000 km). Genetic connectivity was described for Chaetognatha species in the North Atlantic, although that study included only three specimens of *F*. *enflata*, from the SS (n = 2) and the MAB (n = 1) (none from the South and Equatorial Atlantic) [19]. In this former study, the lack of association between diversity and geographic location was attributed to the chaetognaths’ high potential for genetic mixing and/or to a relatively short evolutionary time for the populations to split into genetically distinct lineages [19]. However, in the present investigation all methodological approaches used (network topology, BAPS analysis, AMOVA, Pairwise fixation indices, Migrate-N) depicted a clear population structure. Global fixation index was 0.033 (p [of panmixia] < 0.001). This result unambiguously rejects the null model of panmixia. Crandall et al. [74] found median PhiST for 33 species measured across the Sunda Shelf Barrier (the major phylogeographic barrier between the Indian and Pacific Oceans) to be 0.021. Their simulation of two (large) marine populations diverging in complete allopatry starting 10,000 generations ago with the Last Glacial Maximum yielded median PhiST of 0.023. Most of our secondary pairwise comparisons (Φst) were > 0.030 and 7 out of 15 were significant. Differentiation in marine species is characteristically defined by lower values (when compared with terrestrial species) [73–75]. Ward et al., for example, proposed that differentiation threshold was 0.062 in marine fishes [75]. Later, Waples used this data to highlight that, despite the average was 0.062, the median was 0.020, i.e. much lower [73]. Both authors agreed that the values <0.03 recorded by Ward et al. [87] could be considered indicative of differentiation. Migrate-N results mostly reflected the AMOVA tests. The best MM of 4 populations (MM4.2) defined PR, TA, SPSPA+FN and GS+RA as sites from which migrants are unlikely to interbreed. PR and TA were predicted as independent metapopulations in the AMOVA general tests (Table 2). According to the pairwise comparisons PR was also a distinct group and TA was distinguished from FN. SPSPA was not distinct of FN but was distinct of PR, TA and GS. GS was not significantly distinct of RA (Table 3). The ambiguity scenario posed by the comparison between the first and second best MMs may be attributed to (a) the large variance of Migrate-N marginal likelihoods (as observed by [64]); (b) the medium-low sample size; (c) the mtDNA single locus approach (Crandall et al. [64] found that 37% of mtDNA datasets yielded ambiguous results), or a combination of these factors. Nevertheless it is noteworthy that the first and second models (MM4.2 and MM2.3) are not totally contradictory, since MM2.3 does not distinguish the 2 neritic groups (PR, TA) or the 2 oceanic groups (SPSPA, FN and GS, RA) of MM4.2. Replicating samples over time might allow a better performance in defining the metapopulations of *F*. *enflata* in the TWA.

Altogether the results demonstrate population structure for *F*. *enflata* in the TWA, but important gene fluxes are expected to undergo among some locations.

The flow of this connectivity may be related to the surface and subsurface currents dynamics that circulate in the basin of the tropical Atlantic, and which remained relatively stable since the closure of the Panama Isthmus [76, 77]. The western portion of the Atlantic is mainly controlled by three branches (south, central and north) of the SEC at the surface [78]. The sSEC carries subtropical waters towards the Brazil shelf region, where around 14°S, it bifurcates into the NBUC to the north and in the Brazil Current (BC) to the south [79]. On its course from east to west, the cSEC and nSEC exert strong influence on the region of oceanic seamounts and islands of northeastern Brazil, passing through the SPSPA and FN chain, flowing posteriorly towards the Brazilian coast [80]. Moreover, the SEUC and EUC are subsuperficial systems which contribute with the marine flow in the opposite direction to that of the SEC (west to east). The EUC is located at depths of 50 to 200 m [81], and is fed by the NBC/NBUC system that crosses the Equator [80]. Studies developed in the area report that the currents may effectively contribute to the dispersal of pelagic larvae in several directions, both in neritic and oceanic sites of the TWA, resulting in genetic connectivity patterns among populations [25, 82, 83]. Thus, marine circulation may be favoring *F*. *enflata* gene flow in both directions of the currents, since this species is vertically distributed throughout the entire epipelagic layer.

Available data correlating connectivity to current systems in the TWA are based on species with different life cycles, and investigated by distinct molecular markers. For instance, the fish *C*. *fulva* was described as one population connected by high levels of gene flow among the northeastern and southeastern coasts of Brazil (states of Ceará, Rio Grande do Norte, Bahia and Espírito Santo) and the FN and RA oceanic islands [25]. Similar lineages of the Cephalopoda *Octopus insularis* were also recorded between the northeastern coast (states of Rio Grande do Norte and Pernambuco) and the same oceanic islands evaluated in this study (SPSPA, FN and RA) [83]. In addition, even semi-terrestrial species as the crabs *Johngarthia lagostoma* [84] and *Grapsus grapsus* [85] constitute genetically homogeneous populations between FN and RA; and between SPSPA, FN and RA, respectively. Recently, an individual-based modeling study coupled with a regional hydrodynamic model of the ocean [86] was used to determine the demographic connectivity of reef fishes based on the widespread genus *Sparisoma*, from oceanic islands and the Brazilian continental shelf between 10°N and 23°S. The study showed connectivity amongst FN, RA and coastal areas, but low connectivity between other areas. All these organisms have in common a planktonic phase in their life cycle, during which they might disperse throughout long distances and seemingly establish viable populations in different locations.

Up to now there is no consensus on the lifetime of Chaetognatha. The available investigations based in different methodologies demonstrate that temperature is an important parameter influencing the body size and sexual maturity, reflecting in life cycles with variable durations. Ocean temperature varies according to season, latitude and depth. Therefore, the lifetime of holoplanktonic species is expected to be also influenced by these factors. The life-cycle of *F*. *enflata* was estimated in ~47d in Chile's neritic waters [87]. It is unknown if this estimate resembles the lifetime of the species in tropical environments as those uncovered in the present study. However, a lifetime of this order should allow *F*. *enflata* transiting between oceanic waters and the Brazilian coast. The observation of continuous reproductive cycles over the whole year, a common feature of Chaetognatha species [5], would further contribute to its dispersion in the evaluated areas.

### Guará seamount

GS presented one of the highest haplotype diversity indices detected (Table 1), and shared no haplotypes with any other location (Fig 3), which is exceptional for a marine species. Significant levels of differentiation were recorded between this location and PR, SPSPA and FN (Table 3). Besides, GS was the only area where the haplogroup 2 was not detected, and where haplogroup 1 prevailed (> 90%) (Fig 3B). Interestingly, *F*. *enflata* from GS were more similar to those collected off TA continental shelf (distant ~539 km), than to those from FN (~430 km), or from SPSPA. Such pattern might result from the current circulation among sites. Between TA and GS both surface and subsurface currents flow consistently from the first to the second site (NBC/NBUC system; Fig 4). A water flow also occurs between GS and RA, but there is a retroflection of the surface currents in FN area, what might explain the MM4.2 result, which grouped GS and RA, FN and SPSPA. Consistently there was lower genetic similarity between GS and SPSPA, as well as between TA and FN. Subsurface currents still diverge at 4°S—34°W, with a partial flow eastwards diverging from the NBUC-NBC system, which contributes to feeding the SEUC (Fig 4).

Together, these particularities of the oceanic circulation could originate the significant differences observed between GS and other locations (Table 3).

### Port of Recife

PR was distinguished by being the only area where the haplogroup 2 was dominant (Fig 3B). It also presented the highest and most significant pairwise Φst values when compared to all oceanic locations (Table 3), illustrating the presence of regional characteristics limiting the connectivity and confining the population.

PR is inserted in a semi-closed basin, influenced by the coastal dynamics and bounded by a reef line that runs parallel to the littoral (Fig 1) [88]. This reef is a physical barrier capable of constraining the local water turnover ratio, limiting the flow of planktonic organisms outwards the port basin [89]. Additionally, currents as the NBC and NBUC flow along the external shelf of the region, isolating PR from the ocean circulation. These factors might be acting synergistically, impairing the migration of *F*. *enflata* to the outside and/or inside of the port. If so, PR might be under a structuring process, herein detected using COI gene sequences. This hypothesis deserves further investigation, increasing sample size and including sites outside the reef line.

### Demographic history

Tajima's *D* and Fu's *Fs* estimates were negative for most of the locations. Only TA showed a significant value for Fu's index (p < 0.02—Table 4). In general, significant negative values are associated with demographic expansion events [29, 66] especially in face of high haplotype diversity [90, 91]. Such results also have been attributed to purifying sweeps [92, 93] or directional selection [65]. However, Wares [94] performed a meta-analysis across 1068 COI datasets belonging to 12 taxa, comprising mostly marine species, and found that mean *D* was -0.391 with over a sixth representing significant divergence from null. It might be that negative *D* is commonplace, especially for marine taxa. In general Fu’s *Fs* correlates with *D*, what we did observe, except for 2 localities (SPSPA and RA) where one was positive and the other was negative, but both means were negative. Our results, in light of those abovementioned including embedded citations [94], corroborate the idea that more sophisticated evaluation might be required for demographic reconstruction, including additional genomic data.

## Conclusions

The use of the mtCOI gene allowed to successfully evaluate the diversity and phylogeographic aspects of *F*. *enflata*. We demonstrated clear population structure for it in the TWA. This was outstanding for PR, characterized by specific geographic and dynamic properties, and GS which shared no haplotypes with any other population, a remarkable feature in a marine species. Our results thus challenge the idea of a pure panmitic population, which is the expected standard for hermaphrodite species with long evolutionary history (*F*. *enflata* ~540 Myr).

Marine populations in general were once seen as demographically open, with genetic isolation hard to develop over the long term. However, this idea has been increasingly hard to sustain since the growth of genetic data from varied markers and new tools have been accumulating evidences of multiple levels of geographic structuring. Structured populations of holoplanktonic and widespread crustaceans including copepods, krill and shrimp (*Lucifer hanseni* Nobili) [95–98] testify that the *status quo* thinking needs revaluation. In the case of *F*. *enflata*, future studies should enlarge the sample representativeness and the spatiotemporal span, in order to clarify its metapopulations in the TWA. This work highlights the phylogeography and population genetics of an holoplanktonic species numerically dominant in the TWA, with key role in trophic webs, impacting zoo- and ichthyoplanktonic marine communities.

## Supporting information

S1 Table Identification, geographic location, sampling date and GenBank accession numbers of *Flaccisagitta enflata* analyzed in the present study. (DOCX)

S2 Table Information on the COI sequences used as a reference for the investigation of the evolutionary history of *Flaccisagitta enflata* analyzed in the present study. (DOCX)
