# Correction: Genetic diversity and connectivity of *Flaccisagitta enflata* (Chaetognatha: Sagittidae) in the tropical Atlantic Ocean (northeastern Brazil)

**DOI:** 10.1371/journal.pone.0234679

**Published:** 2020-06-09

**Authors:** The PLOS ONE Staff

There is an error in the Author Contributions for author Lucas Freitas. Lucas Freitas’s roles should be listed as Methodology and Software, not Methodology and Supervision.

There are errors in the Funding statement. The correct Funding statement is as follows: This study is supported by Coordenação de Aperfeiçoamento de Pessoal de Nível Superior (CAPES): Doctoral scholarship to DCMM and research grant to APBM; Conselho Nacional de Desenvolvimento Científico e Tecnológico (CNPq), Project: Plankton community in the Saint Peter and Saint Paul Archipelago and its association with physical mechanisms: vertical distribution of diversity and productivity (Process 405499/2012-4). Institution: Universidade Federal de Pernambuco; Departamento de Oceanografia to SNL; “Acoustics along the Brazilian Coast 2” (ABRAÇOS 2). Institution: French Oceanographic Fleet—DOI: 10.17600/17004100 to APAB—Institut de Recherche pour le Développement, Université Montpellier, France; Departamento de Pesca e Aquicultura, Universidade Federal Rural de Pernambuco; LMI TAPIOCA (Tropical Atlantic Interdisciplinary laboratory on physical, biogeochemical ecological and human dynamics); and PADDLE project (funding by the European Union’s Horizon 2020 research and innovation programme—grant agreement No. 73427). The funders had no role in study design, data collection and analysis, decision to publish, or preparation of the manuscript.

The publisher apologizes for the errors.
