# Supplementary material for: Genetic diversity and connectivity of Flaccisagitta enflata (Chaetognatha: Sagittidae) in the tropical Atlantic ocean (northeastern Brazil)
Source: PLoS One. 2020 May 6;15(5):e0231574. doi: 10.1371/journal.pone.0231574 (PMC7202658; doi:10.1371/journal.pone.0231574)
Supplement: S1 Table — (DOCX) [file pone.0231574.s001.docx]

**S1 Table.** Identification, geographic location, sampling date and GenBank accession numbers of *Flaccisagitta enflata* analyzed in the present study.

|  |  |  |  |  |
| --- | --- | --- | --- | --- |
| **Identification** | **Geographic Location** | **Date** | **Latitude Longitude** | **Accession Number** |
| TA1 | Tamandaré | 10-Mar-2016 | 08° 46’ 22'' S 34° 55’ 53'' W | MH244934 |
| TA3 | Tamandaré | 10-Mar-2016 | 08° 46’ 22'' S 34° 55’ 53'' W | MH244935 |
| TA4 | Tamandaré | 10-Mar-2016 | 08° 46’ 22'' S 34° 55’ 53'' W | MH244936 |
| TA7 | Tamandaré | 10-Mar-2016 | 08° 46’ 22'' S 34° 55’ 53'' W | MH244937 |
| TA8 | Tamandaré | 10-Mar-2016 | 08° 46’ 22'' S 34° 55’ 53'' W | MH244938 |
| TA9 | Tamandaré | 10-Mar-2016 | 08° 46’ 22'' S 34° 55’ 53'' W | MH244939 |
| TA10 | Tamandaré | 10-Mar-2016 | 08° 46’ 22'' S 34° 55’ 53'' W | MH244940 |
| TA12 | Tamandaré | 10-Mar-2016 | 08° 46’ 22'' S 34° 55’ 53'' W | MH244941 |
| TA13 | Tamandaré | 10-Mar-2016 | 08° 46’ 22'' S 34° 55’ 53'' W | MH244942 |
| TA14 | Tamandaré | 10-Mar-2016 | 08° 46’ 22'' S 34° 55’ 53'' W | MH244943 |
| TA15 | Tamandaré | 10-Mar-2016 | 08° 46’ 22'' S 34° 55’ 53'' W | MH244944 |
| TA17 | Tamandaré | 10-Mar-2016 | 08° 46’ 22'' S 34° 55’ 53'' W | MH244945 |
| TA18 | Tamandaré | 10-Mar-2016 | 08° 46’ 22'' S 34° 55’ 53'' W | MH244946 |
| TA22 | Tamandaré | 10-Mar-2016 | 08° 46’ 22'' S 34° 55’ 53'' W | MH244947 |
| TA24 | Tamandaré | 10-Mar-2016 | 08° 46’ 22'' S 34° 55’ 53'' W | MH244948 |
| TA26 | Tamandaré | 10-Mar-2016 | 08° 46’ 22'' S 34° 55’ 53'' W | MH244949 |
| PR1 | Port of Recife | 21-Jun-2016 | 08° 3’ 49.42'' S 34°52’11.69'' W | MH244950 |
| PR2 | Port of Recife | 21-Jun-2016 | 08° 3’ 49.42'' S 34°52’11.69'' W | MH244951 |
| PR3 | Port of Recife | 21-Jun-2016 | 08° 3’ 49.42'' S 34°52’11.69'' W | MH244952 |
| PR4 | Port of Recife | 21-Jun-2016 | 08° 3’ 49.42'' S 34°52’11.69'' W | MH244953 |
| PR5 | Port of Recife | 21-Jun-2016 | 08° 3’ 49.42'' S 34°52’11.69'' W | MH244954 |
| PR6 | Port of Recife | 21-Jun-2016 | 08° 3’ 49.42'' S 34°52’11.69'' W | MH244955 |
| PR7 | Port of Recife | 21-Jun-2016 | 08° 3’ 49.42'' S 34°52’11.69'' W | MH244956 |
| PR8 | Port of Recife | 21-Jun-2016 | 08° 3’ 49.42'' S 34°52’11.69'' W | MH244957 |
| PR9 | Port of Recife | 21-Jun-2016 | 08° 3’ 49.42'' S 34°52’11.69'' W | MH244958 |
| PR11 | Port of Recife | 21-Jun-2016 | 08° 3’ 49.42'' S 34°52’11.69'' W | MH244959 |
| PR12 | Port of Recife | 21-Jun-2016 | 08° 3’ 49.42'' S 34°52’11.69'' W | MH244960 |
| PR13 | Port of Recife | 21-Jun-2016 | 08° 3’ 49.42'' S 34°52’11.69'' W | MH244961 |
| PR14 | Port of Recife | 21-Jun-2016 | 08° 3’ 49.42'' S 34°52’11.69'' W | MH244962 |
| PR15 | Port of Recife | 21-Jun-2016 | 08° 3’ 49.42'' S 34°52’11.69'' W | MH244963 |
| PR16 | Port of Recife | 21-Jun-2016 | 08° 3’ 49.42'' S 34°52’11.69'' W | MH244964 |
| PR17 | Port of Recife | 21-Jun-2016 | 08° 3’ 49.42'' S 34°52’11.69'' W | MH244965 |
| PR18 | Port of Recife | 21-Jun-2016 | 08° 3’ 49.42'' S 34°52’11.69'' W | MH244966 |
| PR19 | Port of Recife | 21-Jun-2016 | 08° 3’ 49.42'' S 34°52’11.69'' W | MH244967 |
| PR20 | Port of Recife | 21-Jun-2016 | 08° 3’ 49.42'' S 34°52’11.69'' W | MH244968 |
| PR21 | Port of Recife | 21-Jun-2016 | 08° 3’ 49.42'' S 34°52’11.69'' W | MH244969 |
| PR22 | Port of Recife | 21-Jun-2016 | 08° 3’ 49.42'' S 34°52’11.69'' W | MH244970 |
| PR23 | Port of Recife | 21-Jun-2016 | 08° 3’ 49.42'' S 34°52’11.69'' W | MH244971 |
| PR24 | Port of Recife | 21-Jun-2016 | 08° 3’ 49.42'' S 34°52’11.69'' W | MH244972 |
| PR25 | Port of Recife | 21-Jun-2016 | 08° 3’ 49.42'' S 34°52’11.69'' W | MH244973 |
| PR26 | Port of Recife | 21-Jun-2016 | 08° 3’ 49.42'' S 34°52’11.69'' W | MH244974 |
| PR27 | Port of Recife | 21-Jun-2016 | 08° 3’ 49.42'' S 34°52’11.69'' W | MH244975 |
| PR28 | Port of Recife | 21-Jun-2016 | 08° 3’ 49.42'' S 34°52’11.69'' W | MH244976 |
| PR29 | Port of Recife | 21-Jun-2016 | 08° 3’ 49.42'' S 34°52’11.69'' W | MH244977 |
| PR30 | Port of Recife | 21-Jun-2016 | 08° 3’ 49.42'' S 34°52’11.69'' W | MH244978 |
| PR32 | Port of Recife | 21-Jun-2016 | 08° 3’ 49.42'' S 34°52’11.69'' W | MH244979 |
| PR33 | Port of Recife | 21-Jun-2016 | 08° 3’ 49.42'' S 34°52’11.69'' W | MH244980 |
| PR34 | Port of Recife | 21-Jun-2016 | 08° 3’ 49.42'' S 34°52’11.69'' W | MH244981 |
| PR35 | Port of Recife | 21-Jun-2016 | 08° 3’ 49.42'' S 34°52’11.69'' W | MH244982 |
| PR36 | Port of Recife | 21-Jun-2016 | 08° 3’ 49.42'' S 34°52’11.69'' W | MH244983 |
| PR38 | Port of Recife | 21-Jun-2016 | 08° 3’ 49.42'' S 34°52’11.69'' W | MH244984 |
| PR40 | Port of Recife | 21-Jun-2016 | 08° 3’ 49.42'' S 34°52’11.69'' W | MH244985 |
| SPSPA4 | Saint Peter and Saint Paul’s Archipelago | 11-Jan-2017 | 00° 55' N 29° 21.17' W | MH244986 |
| SPSPA5 | Saint Peter and Saint Paul’s Archipelago | 11-Jan-2017 | 00° 54' N 29° 20.55' W | MH244987 |
| SPSPA6 | Saint Peter and Saint Paul’s Archipelago | 11-Jan-2017 | 00° 55' N 29° 21.17' W | MH244988 |
| SPSPA7 | Saint Peter and Saint Paul’s Archipelago | 11-Jan-2017 | 00° 55' N 29° 21.17' W | MH244989 |
| SPSPA8 | Saint Peter and Saint Paul’s Archipelago | 11-Jan-2017 | 00° 55' N 29° 21.17' W | MH244990 |
| SPSPA9 | Saint Peter and Saint Paul’s Archipelago | 11-Jan-2017 | 00° 55' N 29° 21.17' W | MH244991 |
| SPSPA10 | Saint Peter and Saint Paul’s Archipelago | 11-Jan-2017 | 00° 55' N 29° 21.17' W | MH244992 |
| SPSPA11 | Saint Peter and Saint Paul’s Archipelago | 11-Jan-2017 | 00° 56.99' N 29° 20.44' W | MH244993 |
| SPSPA12 | Saint Peter and Saint Paul’s Archipelago | 11-Jan-2017 | 00° 54' N 29° 20.55' W | MH244994 |
| SPSPA15 | Saint Peter and Saint Paul’s Archipelago | 11-Jan-2017 | 00° 54' N 29° 20.55' W | MH244995 |
| SPSPA16 | Saint Peter and Saint Paul’s Archipelago | 11-Jan-2017 | 00° 54' N 29° 20.55' W | MH244996 |
| SPSPA17 | Saint Peter and Saint Paul’s Archipelago | 11-Jan-2017 | 00° 54' N 29° 20.55' W | MH244997 |
| SPSPA18 | Saint Peter and Saint Paul’s Archipelago | 11-Jan-2017 | 00° 54' N 29° 20.55' W | MH244998 |
| SPSPA20 | Saint Peter and Saint Paul’s Archipelago | 11-Jan-2017 | 00° 55' N 29° 21.17' W | MH244999 |
| SPSPA21 | Saint Peter and Saint Paul’s Archipelago | 11-Jan-2017 | 00° 55' N 29° 21.17' W | MH245000 |
| SPSPA22 | Saint Peter and Saint Paul’s Archipelago | 11-Jan-2017 | 00° 55' N 29° 21.17' W | MH245001 |
| SPSPA23 | Saint Peter and Saint Paul’s Archipelago | 11-Jan-2017 | 00° 55' N 29° 21.17' W | MH245002 |
| SPSPA25 | Saint Peter and Saint Paul’s Archipelago | 11-Jan-2017 | 00° 55' N 29° 21.17' W | MH245003 |
| SPSPA26 | Saint Peter and Saint Paul’s Archipelago | 11-Jan-2017 | 00° 55' N 29° 21.17' W | MH245004 |
| SPSPA27 | Saint Peter and Saint Paul’s Archipelago | 11-Jan-2017 | 00° 55' N 29° 21.17' W | MH245005 |
| SPSPA28 | Saint Peter and Saint Paul’s Archipelago | 11-Jan-2017 | 00° 55' N 29° 21.17' W | MH245006 |
| SPSPA29 | Saint Peter and Saint Paul’s Archipelago | 11-Jan-2017 | 00° 55' N 29° 21.17' W | MH245007 |
| SPSPA30 | Saint Peter and Saint Paul’s Archipelago | 11-Jan-2017 | 00° 55' N 29° 21.17' W | MH245008 |
| SPSPA32 | Saint Peter and Saint Paul’s Archipelago | 11-Jan-2017 | 00° 55' N 29° 21.17' W | MH245009 |
| SPSPA33 | Saint Peter and Saint Paul’s Archipelago | 11-Jan-2017 | 00° 55' N 29° 21.17' W | MH245010 |
| SPSPA37 | Saint Peter and Saint Paul’s Archipelago | 11-Jan-2017 | 00° 55' N 29° 21.17' W | MH245011 |
| FN2 | Fernando de Noronha Archipelago | 07-Jan-2017 | 03° 49.43' S 32° 24.78' W | MH245012 |
| FN9 | Fernando de Noronha Archipelago | 07-Jan-2017 | 03° 49.43' S 32° 24.78' W | MH245013 |
| FN12 | Fernando de Noronha Archipelago | 07-Jan-2017 | 03° 49.43' S 32° 24.78' W | MH245014 |
| FN13 | Fernando de Noronha Archipelago | 07-Jan-2017 | 03° 49.43' S 32° 24.78' W | MH245015 |
| FN14 | Fernando de Noronha Archipelago | 07-Jan-2017 | 03° 49.43' S 32° 24.78' W | MH245016 |
| FN15 | Fernando de Noronha Archipelago | 07-Jan-2017 | 03° 49.43' S 32° 24.78' W | MH245017 |
| FN16 | Fernando de Noronha Archipelago | 07-Jan-2017 | 03° 49.43' S 32° 24.78' W | MH245018 |
| FN17 | Fernando de Noronha Archipelago | 07-Jan-2017 | 03° 49.43' S 32° 24.78' W | MH245019 |
| FN18 | Fernando de Noronha Archipelago | 07-Jan-2017 | 03° 49.43' S 32° 24.78' W | MH245020 |
| FN23 | Fernando de Noronha Archipelago | 07-Jan-2017 | 03° 49.43' S 32° 24.78' W | MH245021 |
| FN24 | Fernando de Noronha Archipelago | 26-Jan-2017 | 03° 48.45' S 32° 24.20' W | MH245022 |
| FN25 | Fernando de Noronha Archipelago | 26-Jan-2017 | 03° 48.45' S 32° 24.20' W | MH245023 |
| FN28 | Fernando de Noronha Archipelago | 26-Jan-2017 | 03° 47.29' S 32° 23.9' W | MH245024 |
| FN29 | Fernando de Noronha Archipelago | 26-Jan-2017 | 03° 47.29' S 32° 23.9' W | MH245025 |
| FN30 | Fernando de Noronha Archipelago | 26-Jan-2017 | 03° 47.36' S 32° 23.25' W | MH245026 |
| FN32 | Fernando de Noronha Archipelago | 26-Jan-2017 | 03° 48.45' S 32° 24.20' W | MH245027 |
| FN36 | Fernando de Noronha Archipelago | 26-Jan-2017 | 03° 47.29' S 32° 23.9' W | MH245028 |
| FN37 | Fernando de Noronha Archipelago | 26-Jan-2017 | 03° 47.29' S 32° 23.9' W | MH245029 |
| FN38 | Fernando de Noronha Archipelago | 26-Jan-2017 | 03° 47.29' S 32° 23.9' W | MH245030 |
| GS4 | Guará seamount | 06-May-2017 | 04° 5’ 24.79'' S 36° 18’ 03.42'' W | MH245031 |
| GS8 | Guará seamount | 06-May-2017 | 04° 5’ 24.79'' S 36° 18’ 03.42'' W | MH245032 |
| GS9 | Guará seamount | 06-May-2017 | 04° 5’ 24.79'' S 36° 18’ 03.42'' W | MH245033 |
| GS10 | Guará seamount | 06-May-2017 | 04° 5’ 24.79'' S 36° 18’ 03.42'' W | MH245034 |
| GS11 | Guará seamount | 06-May-2017 | 04° 5’ 24.79'' S 36° 18’ 03.42'' W | MH245035 |
| GS14 | Guará seamount | 06-May-2017 | 04° 5’ 24.79'' S 36° 18’ 03.42'' W | MH245036 |
| GS15 | Guará seamount | 06-May-2017 | 04° 5’ 24.79'' S 36° 18’ 03.42'' W | MH245037 |
| GS19 | Guará seamount | 06-May-2017 | 04° 5’ 24.79'' S 36° 18’ 03.42'' W | MH245038 |
| GS20 | Guará seamount | 06-May-2017 | 04° 5’ 24.79'' S 36° 18’ 03.42'' W | MH245039 |
| GS22 | Guará seamount | 06-May-2017 | 04° 5’ 24.79'' S 36° 18’ 03.42'' W | MH245040 |
| GS23 | Guará seamount | 06-May-2017 | 04° 5’ 24.79'' S 36° 18’ 03.42'' W | MH245041 |
| GS25 | Guará seamount | 06-May-2017 | 04° 5’ 24.79'' S 36° 18’ 03.42'' W | MH245042 |
| RA6 | Rocas Atoll | 02-May-2017 | 03° 45' 30.84'' S 34° 00' 5.42'' W | MH245043 |
| RA11 | Rocas Atoll | 02-May-2017 | 03° 45' 30.84'' S 34° 00' 5.42'' W | MH245044 |
| RA12 | Rocas Atoll | 02-May-2017 | 03° 45' 30.84'' S 34° 00' 5.42'' W | MH245045 |
| RA15 | Rocas Atoll | 02-May-2017 | 03° 45' 30.84'' S 34° 00' 5.42'' W | MH245046 |
| RA20 | Rocas Atoll | 02-May-2017 | 03° 45' 30.84'' S 34° 00' 5.42'' W | MH245047 |
| RA21 | Rocas Atoll | 02-May-2017 | 03° 45' 30.84'' S 34° 00' 5.42'' W | MH245048 |
| RA22 | Rocas Atoll | 02-May-2017 | 03° 45' 30.84'' S 34° 00' 5.42'' W | MH245049 |
|  |  |  |  |  |
|  |  |  |  |  |
