# Supplementary material for: Genetic diversity and connectivity of Flaccisagitta enflata (Chaetognatha: Sagittidae) in the tropical Atlantic ocean (northeastern Brazil)
Source: PLoS One. 2020 May 6;15(5):e0231574. doi: 10.1371/journal.pone.0231574 (PMC7202658; doi:10.1371/journal.pone.0231574)
Supplement: S2 Table — (DOCX) [file pone.0231574.s002.docx]

**S2 Table.** Information on the COI sequences used as a reference for the investigation of the evolutionary history of *Flaccisagitta enflata* analyzed in the present study.

|  |  | |  | | |  | | | | |  |
| --- | --- | --- | --- | --- | --- | --- | --- | --- | --- | --- | --- |
| **Identification** | | **Deposited as** | | | **Current Taxonomy** | | | **Accession Number** | | **Authors** | |
| UCONN:Ch15.1.1 | | *Sagitta enflata* | | | *Flaccisagitta enflata* | | | GQ368399.1 | | Jennings RM, Bucklin A, Pierrot-Bults A. | |
| UCONN:Ch15.1.2 | | *Sagitta enflata* | | | *Flaccisagitta enflata* | | | GQ368400.1 | | Jennings RM, Bucklin A, Pierrot-Bults A. | |
| UCONN:Ch15.2.1 | | *Sagitta enflata* | | | *Flaccisagitta enflata* | | | GQ368401.1 | | Jennings RM, Bucklin A, Pierrot-Bults A. | |
| NIOBZC17 | | *Sagitta hexaptera* | | | *Flaccisagitta hexaptera* | | | JN258017.1 | | Nair VR, Giridharaprabhu R, Xavier F, Nair S. | |
| NIOBZC18 | | *Sagitta hexaptera* | | | *Flaccisagitta hexaptera* | | | JN258018.1 | | Nair VR, Giridharaprabhu R, Xavier F, Nair S. | |
| NIOBZC19 | | *Sagitta hexaptera* | | | *Flaccisagitta hexaptera* | | | JN258019.1 | | Nair VR, Giridharaprabhu R, Xavier F, Nair S. | |
| UCONN:Ch18.1.1 | | *Sagitta marri* | | | *Solidosagitta marri* | | | GQ368412.1 | | Jennings RM, Bucklin A, Pierrot-Bults A. | |
| UCONN:Ch18.1.2 | | *Sagitta marri* | | | *Solidosagitta marri* | | | GQ368413.1 | | Jennings RM, Bucklin A, Pierrot-Bults A. | |
| UCONN:Ch18.1.3 | | *Sagitta marri* | | | *Solidosagitta marri* | | | GQ368414.1 | | Jennings RM, Bucklin A, Pierrot-Bults A. | |
| UCONN:Ch16.1.1 | | *Sagitta helenae* | | | *Sagitta helenae* | | | GQ368402.1 | | Jennings RM, Bucklin A, Pierrot-Bults A. | |
| UCONN:Ch16.2.1 | | *Sagitta helenae* | | | *Sagitta helenae* | | | GQ368403.1 | | Jennings RM, Bucklin A, Pierrot-Bults A. | |
| UCONN:Ch16.3.1 | | *Sagitta helenae* | | | *Sagitta helenae* | | | GQ368404.1 | | Jennings RM, Bucklin A, Pierrot-Bults A. | |
| UCONN:Ch22.1.1 | | *Sagitta bipunctata* | | | *Sagitta bipunctata* | | | GQ368396.1 | | Jennings RM, Bucklin A, Pierrot-Bults A. | |
| UCONN:Ch22.1.2 | | *Sagitta bipunctata* | | | *Sagitta bipunctata* | | | GQ368397.1 | | Jennings RM, Bucklin A, Pierrot-Bults A. | |
| UCONN:Ch22.2.1 | | *Sagitta bipunctata* | | | *Sagitta bipunctata* | | | GQ368398.1 | | Jennings RM, Bucklin A, Pierrot-Bults A. | |
|  | |  | |  | | |  | |  | | |
